# Insights on the mechanism of flecainide in catecholaminergic polymorphic ventricular tachycardia

**DOI:** 10.25122/jml-2023-0191

**Published:** 2023-08

**Authors:** Ross Davidson, Maria Medeiros

**Affiliations:** 1 Department of Medicine, King’s College Hospital, Denmark Hill, London

**Keywords:** catecholaminergic polymorphic ventricular tachycardia, flecainide, antiarrhythmic drugs, ryanodine receptor

## Abstract

Catecholaminergic polymorphic ventricular tachycardia (CPVT) is an inherited arrhythmia syndrome characterized by defective cardiac ryanodine receptor (RyR2) calcium release during times of adrenergic stimulation, resulting in bidirectional or polymorphic ventricular tachycardia. Flecainide is a class 1c anti-arrhythmic drug that has demonstrated therapeutic efficacy in treating CPVT. However, its mechanism of action remains disputed. One group proposes a direct effect of flecainide on RyR2-mediated calcium release, while another proposes an indirect effect via sodium channel blockade and modulation of intracellular calcium dynamics. In light of recent studies, this commentary aims to explore and discuss the evidence base for these potential mechanisms.

## INTRODUCTION

Catecholaminergic polymorphic ventricular tachycardia (CPVT) is an inherited arrhythmia syndrome characterized by adrenergically mediated polymorphic or bidirectional ventricular tachycardia, leading to syncope and sudden cardiac death. The most common genetic mutations resulting in CPVT are found within genes responsible for coding the cardiac ryanodine receptor (RyR2). These are usually inherited in an autosomal dominant manner and are termed CPVT1. A recessive form (CPVT2) is caused by gene mutations for cardiac calsequestrin. CPVT is linked to seven genes with autosomal dominant (RYR2, CALM1, CALM2, CALM3) or autosomal recessive (CASQ2, TRDN, TECRL) inheritance [1].

This results in a transient inward current, perpetuating the onset of malignant arrhythmias via delayed afterdepolarizations (DAD) and triggered activity. The first-line drug therapy for CPVT is β-adrenergic receptor blockers. However, some patients are refractory and experience persistent tachyarrhythmias. The class 1c antiarrhythmic drug flecainide, a known inhibitor of the sodium current (INa), has demonstrated effectiveness in treating CPVT; however, the mechanism responsible for the therapeutic efficacy of flecainide in CPVT remains disputed.

Gaining a comprehensive mechanistic understanding of the clinical efficacy of flecainide in CPVT is critical for cases of arrhythmias that may share similar clinical presentation but differ significantly in their underlying mechanisms. Furthermore, abnormalities in calcium handling, including defective RyR2 functioning, are implicated in several other diseases of significant global burden, including muscular dystrophy, malignant hyperthermia, and Alzheimer’s disease. Consequently, RyR2 is a major therapeutic target, and the development of new drugs targeting RyR2 carries significant clinical potential for treating CPVT and other diseases.

### Review of mechanisms

#### Direct inhibition of RyR2

Hilliard *et al*. [2] compared the effects of flecainide and tetracaine, a known inhibitor of RyR2, on SR Ca2+ spark and wave production in an intact mouse model of CPVT. They proposed a unique mechanism in which flecainide directly inhibits RyR2 in its open state, leading to a reduction in arrhythmogenic Ca2+ waves.

In a recent study, Kryshtal *et al*. [3] investigated flecainide’s antiarrhythmic effects in membrane-permeabilized calsequestrin knockout mouse cardiomyocytes. Flecainide was able to suppress RyR2 Ca2+ release even when the effects of INa were eliminated through blockade with tetrodotoxin. Additionally, INa blockade alone was insufficient to prevent the onset of ventricular tachycardia. The authors concluded that flecainide’s mechanism of action in CPVT is principally via direct RyR2 inhibition.

#### INa mediated reduction in DAD

Other studies proposed contrasting mechanisms explaining wave suppression. Liu *et al*. [4] examined the effect of flecainide on RyR2R4496C+/− myocytes. Flecainide did not impact the amplitude of Ca2+ release from the SR. They proposed that flecainide exerted its anti-arrhythmic effect in CPVT through an INa-mediated increase in the threshold for DAD-induced triggered activity.

#### INa mediated reduction in cytosolic Ca2+ concentration via NCX

In adult rat cardiomyocytes, Sikkel *et al*. [5] conducted imaging studies of spontaneous sarcoplasmic reticulum (SR) Ca2+ release events. Flecainide could only reduce Ca2+ waves when INa was in the active state. Additionally, their data suggested that flecainide significantly increased NCX-mediated Ca2+ efflux. Hence, a novel mechanism was proposed, in which flecainide blockade of INa resulted in increased efflux of Ca2+ via NCX, hence reducing the concentration of Ca2+ in the vicinity of RyR2, thereby reducing the likelihood of arrhythmia via triggered activity and DAD.

Bannister *et al*. [6] investigated the effects of flecainide on cationic flux through human RyR2 incorporated into phospholipid bilayers. Flecainide inhibited ion flux through RyR2 by reducing its open-state probability in the cytosolic-luminal direction but not in the physiologically relevant luminal-cytosolic direction. Furthermore, flecainide only depressed the essential charge compensating K+ counter-current flow through RyR2 by 15% at maximal concentrations, suggesting a minimal modulation of counter-current magnitude at concentrations where antiarrhythmic effects are witnessed. The authors, therefore, concluded that flecainide’s effect in CPVT was likely via modulation of INa.

### Causes of study divergency

Several factors should be considered to account for the varying results of flecainide on SR Ca2+ release via RyR2, which suggest a direct effect in some studies [2, 3] and no effect in others [4-6]. Smith and MacQuaide [7] highlighted the presence of significant variation in baseline Ca2+ sparks and waves between the conflicting studies, potentially due to difficulty in controlling luminal Ca2+ concentration in lightly buffered solutions. Furthermore, β-escin or saponin used in the studies can cause differing levels of permeabilization depending on concentration and duration of exposure. Additionally, Hwang *et al*. [8] demonstrated that flecainide’s antiarrhythmic effects in single cells are reduced in Ca2+ overload. Ca2+ wave inhibition required flecainide application for 10 minutes; however, Sikkel *et al*. [5] only applied it for 5 minutes. Hence, the degree of Ca2+ overload and incubation time are additional factors proposed to explain the divergent results in the literature.

Yang *et al*. [9] used in silico mutagenesis to construct a model of CPVT and then utilized a computational modeling approach to predict drug mechanisms via simulation. Interactions with the Na+ channel alone appeared insufficient to explain the therapeutic efficacy of flecainide in CPVT. However, it has been highlighted that the parameters used to model the flecainide-induced open-state block of RyR2 were obtained from Hilliard *et al*. [2]. As established, flecainide acted as a partial inhibitor of K+ current in the opposite (cytosolic to luminal) direction. Furthermore, the computational models utilized may not accurately represent subcellular Ca2+ dynamics and, hence, any INa-mediated alterations in calcium handling by NCX.

## CONCLUSION

The debate regarding the primary mechanism responsible for the therapeutic efficacy of flecainide in CPVT remains highly disputed and unresolved. Numerous studies propose INa-mediated modulation of intracellular calcium dynamics, while several others propose direct RyR2 inhibition as the primary mechanism. Moving forward, the mechanisms of flecainide in CPVT will increase our knowledge of Ca2+ dysregulation in cardiac myocytes and aid in the development of a more specialized therapeutic approach for CPVT.

**Table 1:** Important experimental studies that propose flecainide mechanisms of action in CPVT

Study	RyR2 Origin	Effect of flecainide	Proposed mechanism of flecainide in CPVT
Hilliard et al. (2010) [2]	1. Intact ventricular CSQ2−/− myocytes. 2. Permeabilized rat ventricular myocytes	1. Reduced Ca2+ wave frequency and decreased spark amplitude 2. Spark mass reduction of 40% 3. Increase in spark frequency	Direct open-state blockade of RyR2
Krystal et al. (2021) [3]	1. Membrane-permeabilized Casq2-/- cardiomyocytes lacking sodium channels 2. Intact Casq2-/- cardiomyocytes	1. Reduced the frequency of spontaneous SR Ca2+ release	
Liu et al. (2011) [4]	1. Permeabilized mouse RyR2-R4496C+/− ventricular myocytes 2. Intact ventricular myocytes	1. Did not reduce the frequency of Ca2+ sparks 2. Increased the threshold for triggered activity	INa dependent modulation of calcium dynamics
Sikkel et al. (2013) [5]	1. Intact rat ventricular myocytes	1. Reduced Ca2+ wave and spark frequency and wave velocity with INa block 2. NCX implicated with INa alterations	
Bannister et al. (2016) [6]	1. Recombinant human RyR2	1. Analogues of flecainide (QX-FL and NU-FL) had minimal effect on cation flux from the luminal to the cytosol via RyR2	

Table includes the origin of RyR2, the flecainide's observed effect, and the proposed mechanism
